# Frequency of Celiac Disease in Patients with Increased Intestinal Gas (Flatulence)

**DOI:** 10.5539/gjhs.v8n6p147

**Published:** 2015-10-26

**Authors:** Mohsen Masoodi, Marjan Mokhtare, Shahram Agah, Mohammad Sina, Mojtaba Soltani-Kermanshahi

**Affiliations:** 1 Gastroenterology Section, Colorectal Research Center, Rasoul-e-Akram Hospital, Iran University of Medical Sciences, Tehran, Iran; 2 Internal Medicine Section, Colorectal Research Center, Rasoul-e-Akram Hospital, Iran University of Medical Sciences, Tehran, Iran; 3 Biostatistics Department, International Branch, Shahid Beheshti University of Medical Sciences, Tehran, Iran

**Keywords:** celiac disease, flatulence, frequency

## Abstract

Excessive flatulence which impairs social performance in patients is one of the common reasons for referrals to gastroenterology clinics. Celiac Disease is a rare but important cause of increased intestinal gas (bloating) and if not diagnosed, patients face complications such as malabsorption, anemia, osteoporosis and even intestinal lymphoma. This study aimed to determine the frequency of Celiac Disease in patients with excessive flatulence. One hundred and fifty patients with a chief complaint of experiencing flatulence more than 15 times a day and lasting for three months were referred to the gastroenterology clinic of Rasoul-e-Akram Teaching Hospital. Serological tests for Celiac Disease, Anti TTG Ab (IgA-IgG) were requested and the patients with positive tests underwent upper GI endoscopy. Biopsies of the second part of the duodenum were then sent to the laboratory. From one hundred and thirty patients who completed the study, 92 (70.7%) were female. Mean age of the patients was 32 ± 13 years. Anti TTG Ab was found in 5 patients (3.85%). Only 2 patients (1.5%) had a documented positive pathology for Celiac Disease. According to the results of this study and other studies, we conclude that Celiac Disease is an uncommon etiology for excessive flatulence but it is of importance to investigate it in excessive flatulence patients.

## 1. Introduction

One of the common reasons for referring patients to GI clinics is increased gas passing from the rectum. In some cases, it disturbs social activities such as attending professional sessions, family gatherings or performing some religious obligations. Natural gas volume that passes daily from the rectum is 500 to 1500 ml (Sugai et al., 2006). The frequency of flatus released in healthy people varies between 10 to 20 times per day although excessive passage of flatus or its foul odor can be uncomfortable for individuals but rarely can be along a serious illness (Prince, 2006). In many cases, structural etiologies cannot be found for flatulence. In other words, flatulence in most cases has been considered as a functional disease.

Evaluation of the patients with flatulence should include history and physical examination. If the patient’s problem is recent, especially with the alarm signs of weight loss, diarrhea, anorexia, abdominal pain (sleep awakening), nocturnal abdominal distension, blood in the stool, fever and vomiting), the problem needs meticulous investigation. These assessments include the stool exam for Giardia and fat, lactose tolerance test, serology and upper GI endoscopy (Leffler et al., 2013; Evans et al., 2011). The major portion of intestinal gas is made of nitrogen, oxygen, carbon dioxide, hydrogen and methane. None of these gases are odorous and the smell of intestinal gas is caused by the presence of sulfur compounds (Kurien et al., 2012).

There are two primary sources for intestinal gas: a) air that is swallowed and b) gas that is produced by intestinal bacteria. In cases such as fast eating, chewing gum and smoking, air swallow is more than usual. Colon contains billions of harmless bacteria as normal flora. Carbohydrates that are digested incompletely by enzymes of the stomach and small intestine are degraded by these gas producing microorganisms. Some conditions can cause increased gas production. For example, in diabetics or scleroderma patients, reduction of mobility of small intestine leads to bacterial overgrowth and an increase in gas production. In other diseases such as Celiac Disease and Short Bowel Syndrome, absorption of carbohydrates diminishes and therefore, production of gas increases (Green & Cellier, 2007; Pais, Duerksen, Pettigrew, & Bernstein, 2008).

Celiac Disease (CD) is a common cause of malabsorption. It is a chronic immune-based enteropathy triggered by dietary Gluten (a protein in wheat, barley, and rye) in genetically predisposed individuals and resolves with the exclusion of gluten from diet (Chomeili et al., 2011). Its approximate incidence is one out of every 113 people in United States (Longo, Fauci, Kasper, & Hauser, 2011). CD is known for Iceberg pattern so a small number of individuals have classic symptoms and manifestations of the disease. The age of onset can be from birth to the eighth decade of life (Shah et al., 2000). Many patients have manifestations that are not clearly related to intestinal malabsorption such as anemia, osteoporosis, neurological symptoms (atypical Celiac Disease). An even larger group is asymptomatic and is detected based on abnormal small bowel histopathology and serology and are referred to as Silent Celiac Disease. There is a clear association between Celiac Disease with Gliadin (a component of gluten) in wheat (Cammarota et al., 2004). Almost all patients with Celiac Sprue are HLA DQ2 and HLA DQ8 positive. Absence of HLA DQ2/DQ8 excludes diagnosis of celiac sprue (Hurlstone & Sanders, 2003).

Evidence suggests that the sero prevalence of CD defined as high levels of anti-tTGA and anti-endomysium antibodies has significantly increased in the last decades at a rate that cannot be attributed to the genetic drive. The increase is coherent with the hygiene hypothesis, which states that a decrease in exposure of the immune system to microbes and external antigens is responsible for the rise in autoimmune and atopic reactions (Sarno, Discepolo, Troncone, & Auricchio, 2015).

Consumption of gluten in genetically susceptible individuals (the enzyme tissue transglutaminase plays the role of auto antibody) leads to the cellular immune response and ultimately damages the intestinal mucosa (especially the Proximal part). The prevalence in first- degree relatives of the patients is 10%. Celiac Disease can include classic symptoms of mal absorption syndrome such as bloating, chronic diarrhea with or without steatorrhea, lactose intolerance or lack of specific micronutrients such as iron deficiency anemia (Lo, Guelrud, Essenfeld, & Bonis, 2007).

Most sensitive and specific tests are Anti Endomysial Ab and Anti TTG, IgA type. Observed classic changes in the duodenal specimen are limited to mucosa and include (Appendix 1):

1) An increase in the number of intra-epithelial lymphocytes.
2) Absence or reduction of villus height with Crypts and epithelial hyperplasia.
3) Cuboidal appearance and nuclei that regularly are not placed in the basal of surface epithelial cells.
4) Increase in lymphocytes and plasma cells in the lamina properia (Green & Cellier, 2007; Kagnoff, 2007; Schulzke, Tröger, & Amasheh, 2009).

However, the presence of a characteristic histologic appearance that reverts toward normal state following the initiation of gluten-free diet establishes the diagnosis of celiac (Rubin, Brandborg, Phelps, & Taylor Jr, 1960).

According to the above-mentioned subjects in this study, we intend to investigate the frequency of celiac disease in patients complaining of increased intestinal gas who have been referred to GI clinic of Rasoul-e-Akram Hospital in Tehran. No similar study was found in the literature that examines celiac disease in patients with flatulence, therefore, this study is substantial in this respect.

## 2. Method

This descriptive cross-sectional study has been conducted on patients with chief complaint of increased intestinal gas passing of more than 15 times a day during the last three months in Rasoul-e-Akram Teaching Hospital affiliated with Iran University of Medical Sciences. One hundred and fifty of such patients referred to the gastroenterology clinic were scrutinized for 6 months. Twenty Patients who were not willing to do testing or endoscopy were excluded from the study and finally, 130 patients completed the study. At first a questionnaire was filled in for each patient that included demographic data, possible associated symptoms (weight loss, abdominal pain, abdominal distension, etc.) and importatnt habits (hookah use and smoking, chewing gum, consumption of sodas, etc.). Then Anti TTG IgA and IgG test was checked (Measurement of serum IgA AntiTTG is recommended as the initial test in people who do not have concomitant IgA deficiency and IgG Anti TTG can be measured in those who are IgA deficient) (Fasano & Catassi, 2012; Rubio-Tapia et al., 2013). To avoid false positive serology human form of Anti TTG was used. At last, the patients with positive tests underwent upper GI endoscopy to take four cold forceps biopsies at second part of the duodenum and then sent for pathologic exam.

Two skilled pathologists studied tissue samples that were unaware the results of the serological tests. According to Marsh criteria (Appendix1), the duodenal mucosa was normal or revealed changes from mild abnormalities to severe atrophy in the epithelial layer (Leffler et al., 2013; Pais, Duerksen, Pettigrew, & Bernstein, 2008).

The data was then analyzed by means of SPSS software and using chi-square test. Tables of frequency distribution and statistical indexes are presented. Significance level in this study was less than 0.05. Statistical analysis was completely supervised by a Biostatistician.

## 3. Results

In this study 130 patients with excessive flatulence were included. Ninety two patients were female (70.8 %) and 38 (29.2%) were male. Also, the mean age of patients was 32 ± 13 years. Minimum age was 19 years and maximum age was 78. Eighty seven patients (67%) were married and 43 patients (33%) single or divorced. In terms of education, the majority of participants(43 people) had bachelor’s degree (33.07%), and only 8 patients had post-graduate education (6.1%). Also, 33 (25.3%) had not completed high school, 20 patients (15.3%)had high school diploma and 26 (20%) had associate degrees (Table 1).

**Table 1:** Demographic characteristics of patient with flatulence

Percent	Frequency	Variables
Sex		
Male	38	29.2
Female	92	70.8
Marital status		
Married	87	67
Single	43	33
Education level		
Under graduated	33	25.4
Diploma	20	15.4
Associated level	26	20
Bachelor	43	33.1
Post graduate	8	6.1

With respect to occupation, 55 (42.3%) patients were housewives, 20 (15.4%) were self-employed, 35 (26.9%) employees and 20 (15.4%) university or school students. Studying the associated symptoms, revealed diarrhea in12 (9.2%) patients, and abdominal distension in 118 (90.7%). The others symptoms can be seen in Table 2.

**Table 2:** Associated symptoms of patient with flatulence

Percent	Frequency	Associated symptoms
Abdominal distension	118	90.7
Abdominal pain	98	75.3
Feel of burping	66	50.7
Constipation	48	36.9
Intolerance of milk	38	29.2
Heart burn	36	27.6
Diarrhea	12	9.2
Vomiting	8	6.1
Weight loss	4	3

The patients were asked about their daily habits that could be associated with increased intestinal gas, including daily smoking of cigarettes and hookah, chewing gum and consumption of sodas (Table 3).

**Table 3:** Daily habits of patient with flatulence

Percent	Frequency	Variable
Consumption soda pop	58	44.6
Cigarette Smoking	35	26.9
Chewing gum	28	21.5
Hookah Smoking	25	19.2

Five (3.85%) cases had positive Anti TTG (Table 4). Among these, 3 patients were female and 2 male. From five cases of positive serology only two had positive pathology for Celiac, who one of them was reported as Marsh 1 and the other as Marsh 3b. In terms of gender one case was male and the one was female. From 5 patients with positive serology, 40% were male and 60% were female. Among those with negative serology, 28.8 percent were male and 71.2% were female. This difference was not significant (p = 0.38). The mean age of the patients with positive serology was 34 ± 6 and with negative serology was 32 ± 16 years old. This relationship again was not statistically significant (p= 0.124). In terms of education in the positive serology group, 40% of the patients had under high school diploma education, 20% had finished high school, 20% associate e degree and 20% bachelor’s degrees and none of the aforementioned cases had post graduate degrees. In the negative serology, almost 25% were under-high school diploma, 15 % had high school diploma, 20% had associate degree, 33 % had bachelor’s degree and 6% were post graduates. These cases indicate lack of association between serological response and the level of education (p = 0.895). In the statistical analyses conducted, no significant relationship was found between positive serology of Celiac and age, sex, education level and occupation of patients.

**Table 4:** Serologic and pathologic results for patients with celiac disease

Anti TTG	Positive	Percent
IgA	4	3.08
IgG	1	0.76
Biopsy	2	1.5

## 4. Discussion

In this study, frequency of Celiac Disease in patients with chief complaint of flatulence, in terms of serology and pathology, was 3.85% and 1.5%, respectively. Screening Studies in many countries show that the majority of celiac cases remain undiagnosed. A study in Europe showed that the proportion of patients with clinical diagnoses and those who are diagnosed by screening is one in seven. In a study conducted by Tirgar, Malekzadeh, Akbari and Sotoudeh (2004) in Sari, 25% of people who had Celiac Disease complained of increased bloating and flatulence and the prevalence of Celiac was approximately one out of every 120 people in the north of Iran. In Shahbazkhani, et al. (2003) study, from 105 patients with irritable bowel syndrome, 11.4% of patients were serologically positive. Ehsani-Ardakanimd et al. (2013) found that 11% of patients who had celiac complained of increased abdominal gas and bloating (19). Masoodi, Sadeghi, and Moosavi (2007) concluded that 19(12.7%) patients out of 150 ones with IBS had positive serologic test for celiac and 15(10%) of them had histologic evidence of Celiac Disease.

Among the cited reasons for the increase of intestinal gas, Celiac disease was one of the main etiologies which in the absence of timely diagnosis and treatment will lead to serious conditions like lymphoma. Ganji et al. (2014) found that 7.2% of Celiac patients in northeast of Iran had flatulence. In the present study, the finding that 93(70%) patients out of 130 were female may indicate that either flatulence is more prevalent in women compared to men or generally women perceive this complaint more than men. Furthermore, the fact that 75% of patients had a degree above high school diploma may show that literacy is a determining factor for paying adequate attention to self-health.

Understandably, higher consumption of carbonated soft drinks is a factor in increased intestinal gas volume. As mentioned above, conditions such as swallowing a large mouthful of food together drinks, chewing gum, smoking and eating in stressful circumstances exacerbate the level of gas in the stomach and intestines. Therefore, avoiding chewing gums, smoking, stress and consumption of flatulence causing foods, such as cabbage and broccoli, grains and high fiber diet helps reduce intestinal gas.

In our study, it was found that among all 130 participants, anti TTG of only 5 (about 3.85%) were positive. These patients then underwent upper GI endoscopy and biopsy for further investigation. For these 5 patients, the reason for endoscopy was fully explained by the doctor and the informed consent was obtained. From tissue samples, 2cases were positive for Celiac. One case was reported as Marsh1 and the other as Marsh 3b.

Several reasons could be listed for positive serology with negative biopsy results: One reason is that serologic tests have false positives, so it is necessary to repeat the test. Another reason could be a false negative in pathology. It is, therefore, imperative that the pathologist be competent enough to discriminate between positive and negative pathologic changes in the specimens to diagnose Celiac. However, if doubt about the definitive diagnosis of the disease still exists, high gluten diet can be initiated, followed by taking several biopsies from different parts of the duodenum 6 to 12 weeks later. Involvement of the duodenum in celiac disease can be patchy. Therefore, specific staining techniques and high resolution magnification endoscopy can be used in order to take better biopsies for identification of areas with villous atrophy (Kurppa et al., 2009).

In this study, some cases were found whose clinical conditions and family history matched with Celiac but blood tests and biopsies of small intestine were negative. The related reasons can be as follows: 1) the individual may have selective IgA deficiency. So, IgG should be checked in such cases. 2) Diet of such patients may have little gluten (so antibodies do not rise and villous atrophy and other pathological changes are not visible). 3) Serologic tests may be falsely negative (so, duodenal biopsy should be done to confirm diagnosis). 4) The patient may not have Celiac disease and the cause of symptoms or atrophy of villi is something else (e.g. Crohn’s disease, Giardiasis, lymphoma …). 5) HLA DQ2 or DQ8 is positive in more than 99% of patients with Celiac while it is positive in 40% of general population. So the diagnosis of Celiac is almost ruled out if in a person that is suspected of having celiac, these HLAs are negative (Biesiekierski et al., 2011; Hadithi et al., 2007).

## 5. Conclusion

Celiac Disease can have serious long-term consequences, including, anemia, osteoporosis, and malignancy. Diagnosing Celiac is important because a gluten-free diet typically resolves all symptoms and can prevent long-term consequences. However, most patients presenting with flatulence and abdominal symptoms will not have Celiac Disease. By comparing the results of this study and those that have addressed the symptoms of patients with celiac diagnosis, we found that Celiac Disease is a relatively rare etiology for intestinal gas related complaints, but needs proper attention because it is treatable.

A study with larger sample size could be recommended to investigate different etiologies of flatulence such as IBD, IBS, pancreatic insufficiency, lactose intolerance and intestinal bacterial overgrowth.
